# Clinical characteristics and outcomes in adult cystic fibrosis patients with severe lung disease in Porto Alegre, southern Brazil

**DOI:** 10.1186/s12890-020-01223-6

**Published:** 2020-07-16

**Authors:** Guilherme Figueiredo Silva, Nicholas J. Simmonds, Paulo de Tarso Roth Dalcin

**Affiliations:** 1 Programa de Pós-Graduação em Ciências Pneumológicas, UFRGS; Serviço de Pneumologia, HCPA, Porto Alegre, Brazil; 2 Department of Cystic Fibrosis, Royal Brompton Hospital and Imperial College, London, UK; 3 Programa de Pós-Graduação em Ciências Pneumológicas, UFRGS, Porto Alegre, Brazil; 4 Serviço de Pneumologia, HCPA, Porto Alegre, Brazil; 5 Honorary Clinical Fellow in the Adult CF Centre of Royal Brompton Hospital, London, UK; 6 Porto Alegre, Brazil

**Keywords:** Cystic fibrosis, FEV_1_, Advanced lung disease, Survival

## Abstract

**Background:**

Advanced lung disease in adult cystic fibrosis (CF) drives most clinical care requirements. The aim was to evaluate outcome (time to death while in the study) in a cohort of adult CF patients with severe lung disease, and to determine the association among baseline patient characteristics and outcome.

**Methods:**

A retrospective cohort study was performed and clinical records between 2000 and 2015 were reviewed. Severe lung disease was defined as forced expiratory volume in the first second (FEV_1_) < 30% of predicted. Outcomes of all patients, including their date of death or transplantation, were determined till January 1st, 2016. Clinical data were recorded at the entry date.

**Results:**

Among 39 subjects included in the study, 20 (51.3%) died, 16 (41.0%) underwent bilateral lung transplantation, and 3 were alive at the end of the study period. Two variables were independently associated with death: body mass index (BMI ≥ 18.5 kg/m^2^) (HR = 0.78, 95% CI = 0.64–0.96 and *p* = 0.017) and use of tobramycin inhalation therapy (HR = 3.82, 95% CI = 1.38–10.6 and *p* = 0.010). Median survival was 37 (95% CI = 16.4–57.6) months. The best cut-off point for BMI was 18.5 kg/m^2^. Median survival in patients with BMI < 18.5 kg/m^2^ was 36 months (95% CI = 18.7–53.3).

**Conclusion:**

Median survival of CF subjects with FEV_1_ < 30% was 37 months. BMI and tobramycin inhalation therapy were independently associated with death. Median survival in patients with BMI < 18.5 kg/m^2^ was significantly lower than in patients with BMI ≥ 18.5 kg/m^2^. The association of tobramycin inhalation with death was interpreted as confounding by severity (use was reserved for advanced lung disease).

## Background

Cystic fibrosis (CF) causes premature death [1]. The prognosis of people with CF has improved considerably over the past decades [2]. Data from a United States registry show that the predicted median survival in CF is currently 47.7 years of age [3]. Consequently, increasing numbers of CF adults reaching middle age or older are predicted for the future [4].

Lung disease is the strongest predictor of death in CF, and forced expiratory volume in 1 second (FEV_1_) is the main parameter used to assess the severity of lung disease in subjects with CF [5]. While a decline in lung function is typical of almost all patients with CF, the rate of decline is highly variable [6].

Although there is significant variation in pulmonary outcomes by centre and birth cohort in both children and adults, there is also variation based on mutation class [3, 7].

Transplantation should be considered for suitable patients with CF who have a 2-year predicted survival of < 50% and who have functional limitations classified as New York Heart Association Class III or IV [8].

Given the shortage of organs, the resulting waiting times, and the unpredictable evolution of end-stage CF, CF patients eligible for lung transplant should be referred to a transplant centre at an appropriate time [9]. An FEV_1_ < 30% of predicted values and/or a rapid decline in FEV_1_ despite optimal conservative treatment (particularly in a female patient), indicates timing of referral in CF. Other indicators for a pretransplant assessment are malnutrition, diabetes, frequent exacerbations and/or an increasing need for intravenous antibiotic therapy, recurrent or massive haemoptysis, relapsing or complicated pneumothorax, or the need for intensive care unit admission [8, 9].

A significant number of CF patients with severe lung disease (FEV_1_ < 30% of predicted) refuse or do not meet the criteria for lung transplant. On the other hand, the demand of lung transplantation continues to exceed the supply of suitable donor organs and the median waiting time for lung transplantation has increased [10]. Consequently, CF patients with advanced lung disease will still require clinical care of the adult CF team. Also, with recent advances in the treatment of CF, patients with advanced pulmonary disease are living longer [11, 12]. Survival with advanced pulmonary disease presents new questions and potential problems which are still being formulated. So, it is important to perform a more detailed study about the characteristics and outcomes of this subgroup of patients.

The current study was therefore designed to evaluate outcome (time to death while in the study) of a cohort of adult CF patients with severe lung disease, seen at the Adult CF Centre of Hospital de Clínicas de Porto Alegre (HCPA), and to determine the association among baseline patient characteristics and outcome.

## Methods

### Study design and population

This was a retrospective cohort study in which chart review was conducted to identify patients with CF diagnosed with severe lung disease, evaluated, or monitored at the Adult CF Centre of HCPA (Porto Alegre, Brazil). Clinical records of Adult CF Program between 2000 and 2015 were reviewed. A cohort of those individuals with severe lung disease defined as FEV_1_ < 30% of predicted was analysed.

Hospital de Clínicas de Porto Alegre (HCPA) is a tertiary care teaching hospital located in Porto Alegre, the capital of the state of Rio Grande do Sul, in southern Brazil. The Adult CF Centre of HCPA is one of the largest adult CF centres in Brazil.

The diagnosis of CF should be based on clinical features and a positive sweat chloride (> 60 mmol/L) or, in cases with a borderline sweat test result, the presence of a known disease-causing mutation on each cystic fibrosis transmembrane conductance regulator (CFTR) gene [13].

For this survey, we reviewed our CF data base and identified all patients who were, between January 1st, 2000 to December 31st, 2015, 18 years or older (adult CF clinic receive patients 16 years or older). To more specifically look at those patients with FEV_1_ < 30% of predicted, we selected patients who had an FEV_1_ < 30% predicted for the first time. It had to be persistently in this range (defined as more than three subsequent measures < 30% predicted within a year, and who did not have a subsequent value > 30% predicted on more than one occasion. Also, patients had to be evaluated when clinically stable, had not been hospitalized, and had no changes in their treatment regimen for at least 30 days before assessment. The date at which the patients met these criteria for the first time (FEV_1_ < 30% predicted) was defined as their “entry date” to create a life table for the group.

The outcomes of all patients, including their date of death or transplantation, were followed till January 1st, 2016. This date was defined as “the end of the study period”.

### Study measures and procedures

Data on the following variables were recorded at “the entry date”: entry date, date of birth, sex, ethnicity, educational level, income level monthly, presence of the F508del mutation (homozygous or heterozygous), body mass index (BMI), pancreatic status, CF related diabetes (CFRD), history of pneumothorax, history of haemoptysis, history of previous diagnosis of allergic bronchopulmonary aspergillosis (ABPA), distal intestinal obstruction syndrome (DIOS), CF liver disease, liver transplant, chronic infection with *Pseudomonas aeruginosa*, chronic infection with *Burkholderia cepacia*, chronic infection with methicillin resistant *Staphylococcus aureus* (MRSA), chronic infection with nontuberculous mycobacteria (NTM), pulmonary artery systolic pressure (PASP) estimated by Doppler echocardiography, use of inhaled dornase alfa, use of inhaled colistin, use of inhaled tobramycin, use of oral azithromycin, pulmonary exacerbations in the last year, lung transplantation status (listed, failed or refused).

In this study, pancreatic insufficiency was defined as the use of enzymes and pancreatic sufficiency as no use of enzymes. Chronic infection by *P. aeruginosa*, *B. cepacia* or MRSA was defined as patients having three or more positive isolates during the previous 12 months. CFRD was identified as use of insulin.

### Ethics

The study was approved by the Ethics Committee of Hospital de Clínicas de Porto Alegre and Plataforma Brasil (protocol number 18–0055). Because this was a retrospective study, the Ethics Committee waived the need for informed consent. The authors signed a data use agreement, protecting the confidentiality of patient information. The clinical research complied with international and national standards for clinical study in human (Declaration of Helsinki and Brazilian Governmental regulation – Plataforma Brasil).

### Statistical analysis

Data analysis was performed using the SPSS 22.0 (SPSS, Chicago, Illinois). Descriptive statistics (mean ± SD, range, n, and proportion) were calculated for characteristics of the study sample.

Actuarial survival was determined by the Life Table method. Kaplan-Meier graphs was used to demonstrate survival over time. Patients who underwent lung transplantation were censored at the time of their operation, and patients who were alive were censored on the “the end of the study period”.

Cox proportional hazards regression methods was used to identify risk factors for death while on the study and to determine the association among baseline patient characteristics and outcomes. Time to death while in the study was the primary outcome. Univariate proportional hazards analyses were performed, and Wald chi-square *p* values were calculated. The likelihood ratio method was used to determine hazard ratios (HR), and the HR was used to approximate the relative risk (RR). Covariates with *p* < 0.1 entered into a forward stepwise multivariate Cox regression analysis. A *p* value > 0.10 was the criterion to remove covariates from the model. The non-collinear covariables that reached significance (*p* < 0.1) in the univariate analysis were included in a multivariate Cox regression analysis with enter method. The most significant variable of this multivariate analysis was correlated with the outcome (death) and submitted to the receiver operating curve (ROC) to determine the cut-off point.

### Sample size calculation

We calculated sample size based on the study of Vizza et al. [10] In this study, the actuarial survival rates for the entire cohort were 59% at 3 year. Considering survival rates of 59% at 3-year, margin of error = 16%, confidence level = 95%, 37 subjects would be needed in the study to show a meaningful survival curve.

## Results

Between January 2000 to December 2015, 152 adult CF patients were evaluated, or monitored at the Adult CF Centre of HCPA. Out of them, 39 subjects were included in the study. There were 27 (69.2%) male subjects and 37 (94.9%) were Caucasian. The mean age at the entry date was 25.3 ± 8.8 years. The mean FVC was 40.5 ± 10.3% of predicted and the mean FEV_1_ was 24.4% ± 3.9 of predicted (Tables 1 and 2).

**Table 1:** Characteristics of the patients at the entry date and associations with case status (death)

	All	Death	Survival	Transplant	HR (95% CI)	***p***-values
**Subjects (n)**	**39**	**20 (51.3)**	**3 (7.7)**	**16 (41.0)**		
Sex, n (%)					0.84 (0.30–2.35)	0.743
Female	12 (30.8)	5 (25.0)	0 (0.0)	7 (43.8)		
Male	27 (69.2)	15 (75.0)	3 (100.0)	9 (56.3)		
Age (years)	25.3 ± 8.8	23.6 ± 8.7	41.7 ± 5.5	24.4 ± 6.1	0.98 (0.92–1.04)	0.456
Ethnicity – Caucasian (yes)	37 (94.9)	19 (51.4)	3 (8.1)	15 (40.5)	0.19 (0.02–1.63)	0.129
Age at diagnosis (years)	7.0 (11.8)	4.8 (7.8)	13.0 (12.5)	7 (16.2)	0.98 (0.93–1.04)	0.560
Educational level, n (%)						
≤ 8 years	7 (17.9)	6 (30.0)	0 (0)	1 (6.3)	–	0.639
> 8 years and < higher education	21 (53.8)	11 (55.0)	1 (33.3)	9 (56.3)	1.34 (0.32–5.6)	0.685
≥ higher education	11 (28.2)	3 (15.0)	2 (66.7)	6 (37.5)	0.81 (0.21–3.08)	0.760
Income level monthly, n (%)						
≤ US$ 231 (≤ 3 BMW)	16 (41.0)	11 (55.0)	0 (0)	5 (31.3)	–	0.270
> US$ 231 to US$ 693 (> 3 to 10 BMW)	12 (30.8)	4 (20.0)	2 (66.7)	6 (37.5)	1.45 (0.50–1.45)	0.500
> US$ 693 (> 10 BMW)	11 (28.2)	3 (25.0)	1 (33.3)	5 (31.3)	0.42 (0.08–2.28)	0.313
BMI (kg/m^2^), mean ± SD	18.6 ± 2.7	17.5 ± 2.2	22.6 ± 0.5	19.3 ± 2.6	0.78 (0.64–0.96)	0.017
F508del mutation						
Homozygous (yes)	16 (41.0)	9 (45.0)	2 (66.7)	5 (31.3)	1.10 (0.44–2.72)	0.843
Heterozygous (yes)	12 (30.8)	6 (30.0)	1 (33.3)	5 (31.3)	1.07 (0.41–2.81)	0.886
Pancreatic insufficiency (yes)	35 (89.7)	20 (100.0)	3 (100.0)	12 (75.0)	0.04 (0.00–11.4)	0.261
CFRD (yes)	16 (41.0)	9 (45.0)	1 (33.3)	6 (37.5)	0.78 (0.31–1.94)	0.590
Any pneumothorax (yes)	8 (20.5)	6 (30.0)	0 (0.0)	2 12.5)	0.48 (0.18–1.29)	0.145
Major haemoptysis > 100 mL (yes)	12 (30.8)	6 (30.0)	1 (33.3)	5 (31.3)	1.44 (0.52–3.99)	0.489
Bronchial artery embolization (yes)	8 (20.5)	3 (15.0)	1 (33.3)	4 (25.0)	2.37 (0.55–10.26)	0.250
CF liver disease (yes)	18 (46.2)	12 (60.0)	1 (33.3)	5 (31,3)	0.53 (0.22–1.30)	0.164
Liver transplant (yes)	1 (2.6)	1 (5.0)	0 (0.0)	0 (0.0)	0.29 (0.04–2.30)	0.241
ABPA (yes)	8 (20.5)	3 (15.0)	0 (0.0)	5 (31.3)	1.94 (0.56–6.70)	0.296
DIOS (yes)	2 (5.1)	1 (5.0)	0 (0.0)	1 (6.3)	1.82 (0.24–13.85)	0.561
*P. aeruginosa* (yes)	35 (89.7)	19 (95.0)	3 (100.0)	13 (81.3)	0.29 (0.04–2.21)	0.234
*S. aureus* (yes)	30 (76.9)	17 (85.0)	2 (66.7)	11 (68.8)	0.52 (0.15–1.78)	0.295
MRSA (yes)	19 (48.7)	13 (65.0)	0 (0.0)	6 (37.5)	0.54 (0.21–1.35)	0.185
*B. cepacia* (yes)	18 (46.2)	10 (50.0)	0 (0.0)	8 (50.0)	1.13 (0.45–2.83)	0.799
NTM (yes)	1 (2.6)	0 (0.0)	0 (0.0)	1 (6.3)		
Entry date in the study					1.11 (0.44–2.81)	0.827
≤ 2007	11 (28.2)	7 (35.0)	0 (0.0)	4 (25.0)		
> 2007	28 (71.8)	13 (65.0)	3 (100.0)	12 (75.0)		

Data are presented as n (%), median ± standard deviation or median (interquartile range). Hazard ratios (HRs) were derived from Cox regression models. *CI* confidence interval, *BMW* Brazilian minimum wage, *BMI* body mass index, *CFRD* cystic fibrosis related diabetes, *ABPA* allergic bronchopulmonary aspergillosis, *DIOS* distal Intestinal obstruction syndrome, *MRSA* methicillin-resistant *Staphylococcus aureus*, *NTM* non-tuberculous mycobacteria

**Table 2:** Pulmonary function tests, 6-min walk distance and pulmonary artery systolic pressure estimated by echocardiography at the entry date and associations with case status (death)

	All	Death	Survival	Transplant	HR (95% CI)	***p***-values
**Subjects (n)**	**39**	**20 (51.3)**	**3 (7.7)**	**16 (41.0)**		
**FVC (% predicted)**	**40.5 ± 10.3**	**42.6 ± 12.9**	**35.7 ± 4.1**	**38.9 ± 8.4**	**1.00 (0.96–1.05)**	**0.837**
**FEV**_**1**_**(% predicted)**	**24.4 ± 3.9**	**24.3 ± 4.2**	**23.3 ± 4.6**	**24.8 ± 3.7**	**0.95 (0.84–1.07)**	**0.362**
**FEV**_**1**_**/FVC (%)**	**54.7 ± 9.4**	**53.6 ± 10.5**	**55.4 ± 3.5**	**55.9 ± 8.9**	**0.98 (0.93–1.04)**	**0.507**
**SpO**_**2**_**(%)**	**91.6 ± 4.3**	**92.7 ± 3.3**	**87.3 ± 4.5**	**91.2 ± 5.1**	**1.03 (0.91–1.2)**	**0.629**
**6-MWT (m)**	**479.9 ± 104.8**	**481.7 ± 115.3**	**514.0 ± 51.9**	**471.6 ± 103.9**	**0.99 (0.99–1.00)**	**0.840**
**PASP ≥ 35 mmHg (yes)**	**15 (38.5)**	**6 (30.0)**	**1 (33.3)**	**8 (50.0)**	**2.17 (0.83–5.72)**	**0.116**

Data are presented as n (%) or median ± standard deviation. Hazard ratios (HRs) were derived from Cox regression models. *CI* confidence interval, *FVC* forced vital capacity, *FEV*_*1*_ forced expiratory volume in the first second, *SpO*_*2*_ room air saturation of peripheral oxygen (evaluated by a non-invasive pulse oximeter), *6-MWd* 6-min walk distance, *PASP* pulmonary artery systolic pressure

Twenty subjects (51.3%) died (death outcome), 16 subjects (41.0%) underwent bilateral lung transplantation (censored at the date of transplantation), and 3 subjects were alive and still waiting for lung transplantation at the end of the study period (survival outcome). Among those 20 subjects who died, 14 did not fulfil lung transplantation criteria, 2 refused the lung transplantation referral and 4 died while waiting on waiting list. The time spent on waiting list for those 4 subjects who died while waiting for lung transplantation was 37.5 ± 10.6 months. The time spent on waiting list for those 16 subjects who underwent lung transplantation was 35.3 ± 24.9 months.

Additional descriptive characteristics are provided in Tables 1, 2 and 3. None of these patients were on use of CFTR modulators.

**Table 3:** Therapeutic support at the entry date and associations with case status (death)

	All	Death	Survival	Transplant	HR (95% CI)	***p***-values
**Subjects (n)**	**39**	**20 (51.3)**	**3 (7.7)**	**16 (41.0)**		
Dornase alfa (yes)	39 (100.0)	20 (100.0)	3 (100.0)	16 (100.0)	–	–
Colistin inhalation (yes)	38 (97.4)	20 (100.0)	2 (66.7)	16 (100.0)	0.05 (0.0–472.26)	0.511
Tobramycin inhalation (yes)	19 (48.7)	15 (75.0)	1 (33.3)	3 (18.8)	3.82 (1.38–10.60)	0.010
Azithromycin (yes)	33 (84.6)	16 (80.0)	3 (100.0)	14 (87.5)	1.32 (0.44–4.01)	0.622
Oxygen therapy (yes)	19 (48.7)	10 (50.0)	3 (100.0)	6 (37.5)	0.55 (0.22–1.36)	0.191
Pulmonary Exacerbations in the last year, median IR)	3 (1)	3 (1)	2(0)	3 (3)	0.86 (0.61–1.21	0.392
Hospital admission in the last year (n), median (IR)	2.1 (2.0)	2.0 (2.0)	0 (0.0)	2.0 (1.0)	1.21 (0.91–1.61)	0.198
NIMV in the last year (yes)	6 (15.4)	2 (10.0)	1 (33.3)	3 (18.8)	0.73 (0.17–3.23)	0.692
ICU admission in the last year (yes)	7 (17.9)	2 (10.0)	1 (33.3)	4 (25.0)	0.75 (0.17–3.43)	0.713
Mechanical ventilation (yes)	5 (12.8)	2 (10.0)	1 (33.3)	2 (12.5)	2.33 (0.51–10.8)	0.278

Data are presented as n (%), median ± standard deviation or median (interquartile range). Hazard ratios (HRs) were derived from Cox regression models. *CI* confidence interval, *NIMV* non-invasive mechanical ventilation, *ICU* intensive care unit

The results of univariate Cox proportional hazards regression methods for death while on the study and the association of case status (death) are shown in Table 1 for characteristics of subjects at the entry date; in Table 2 for pulmonary function tests, 6-min walk test (6MWT) distance and PASP estimated by echocardiography at the entry date; in Table 3 for therapeutic support at the entry date. The following variables were significantly associated with case status (death): BMI kg/m^2^ (HR = 0.78, 95% CI = 0.64–0.96 and *p* = 0.017) and use of tobramycin inhalation therapy (HR = 3.82, 95% CI = 1.38–10.6 and *p* = 0.010). In multivariate Cox regression analysis with enter method both variables were associated with case status: BMI kg/m^2^ (HR = 0.82, 95% CI = 0.67–0.99 and *p* = 0.038) and use of tobramycin inhalation therapy (HR = 3.37, 95% CI = 1.20–9.46 and *p* = 0.021).

In ROC curve analysis for BMI, the area under the curve was 0.694 and the best cut-off point selected was 18.5 kg/m^2^.

**Fig. 1:**
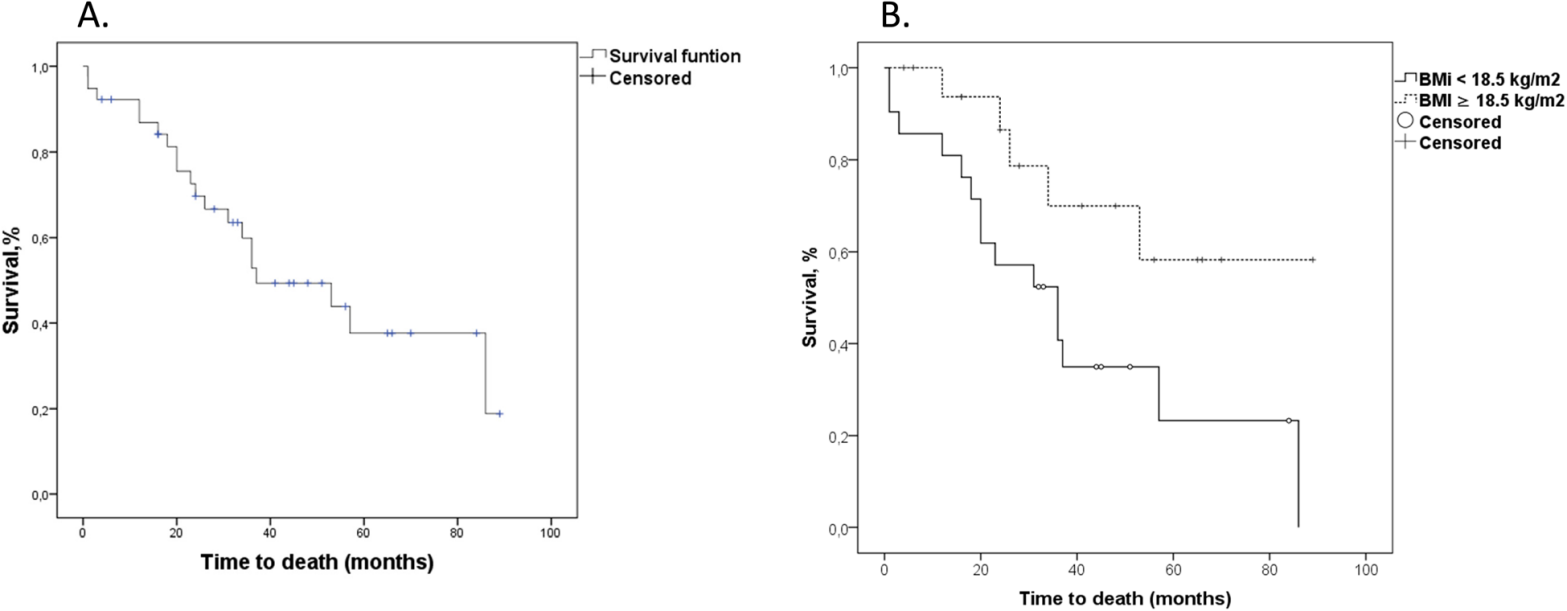
**a** Kaplan-Meier analysis of survival effect in cystic fibrosis patients with FEV1 < 30%. Median survival was 37 (CI = 16.4–57.6) months and mean survival was 50.5 (CI = 39.3–61.6) months. **b** Kaplan-Meier survival functions comparing body mass index (BMI) < 18.5 kg/m2 and BMI ≥ 18.5 kg/m2 in patients with cystic with FEV1 < 30%. Mean survival in patients with BMI < 18.5 kg/m2 = 39.9 (CI 25.8–54.0) months (median survival = 36 monhs, CI = 18.7–53.3) and in patients with BMI ≥ 18.5 kg/m2 median survival = 65.5 (CI 49.2–81.8) months (median survival not measurable), Log-rank test *P* = 0.028

Figure 1a show Kaplan-Meier analysis of survival effect in CF patients with FEV_1_ < 30%. Median survival was 37 (95% CI = 16.4–57.6) months and mean survival was 50.5 (95% CI = 39.3–61.6) months. Figure 1b shows Kaplan-Meier survival functions comparing BMI < 18.5 kg/m^2^ and BMI ≥ 18.5 kg/m^2^. Mean survival in patients with BMI < 18.5 kg/m^2^ was 39.9 (95% CI = 25.8–54.0) months (median survival was 36 motnhs, 95% CI = 18.7–53.3) and in patients with BMI ≥ 18.5 kg/m^2^ mean survival was 65.5 (95% CI = 49.2–81.8) months (median survival not measurable), Log-rank test *P* = 0.028.

## Discussion

In this retrospective cohort study, we demonstrated that the median survival of CF subjects with FEV_1_ < 30% of predicted was 37 months. The main factors associated with time to death while in the study were BMI and tobramycin inhalation therapy. In ROC curve analysis for BMI, the best cut-off point selected was 18.5 kg/m^2^. Mean survival in patients with BMI < 18.5 kg/m^2^ was significantly lower (39.9 months) than in patients with BMI ≥ 18.5 kg/m^2^ (65.5 months). At the end of the study period, 20 (51.3%) died, 16 subjects (41.0%) underwent bilateral lung transplantation, and 3 subjects were alive and still waiting for lung transplantation. There was no difference between patients with entry date in the study after 2007 from those with entry date in 2007 or before.

A measurement of lung function over time to assess disease progression has been the most useful predictor [5]. In 1992, Kerem et al. [14] reported that FEV_1_ < 30% of predicted was associated with a 2-year mortality rate of approximately 50% (38% in men and 56% in women). Milla et al. [15] analysed CF patients who reached and kept an FEV_1_ < 30% predicted. The median survival was 3.9 years and only the rate of decline in percent of predicted FEV_1_ was a significant predictor of the risk of death. Vizza et al. [10] studied a cohort of 146 patients with CF who were listed for lung transplantation. Thirty-seven patients died while waiting, 76 underwent transplantation, and 33 were alive and still waiting. A multivariate model identified shorter six-minute walk distance, higher PASP, and diabetes mellitus as significant risk factors for death on the waiting list.

Augarten et al. [16] evaluated 40 CF patients with FEV_1_ less than 30% predicted. Out of them, 9 patients were transplanted. CF patients with a FEV_1_ < 30% and who did not receive a lung transplant had survived longer than CF patients who did receive a lung transplant (median survival 7.33 vs. 3.49 year, 5-yr survival 73% vs. 29%). Two factors (rate of decline in FEV_1_ values and age < 15 year) were found to influence the mortality rate.

George et al. [12] evaluated 276 adult CF patients in London, whose FEV_1_ was first observed to be less than 30% predicted. Median survival improved from 1.2 years in the 1990 group to 5.3 years in the 2002 group, with a marked improvement in survival from 1994. The use of nebulised recombinant human DNase was significantly associated with a reduced risk of death (HR = 0.59, 95% CI = 0.44–0.79). Significantly increased risks were associated with a BMI under 19 (HR = 1.52, 95% CI = 1.10 to 2.10), long term oxygen therapy (HR = 3.52, 95% CI = 2.49–4.99), and nebulised antibiotics (HR = 1.84, 95% CI = 1.05–3.22).

Ramos et al. [11] conducted a retrospective cohort study using the CF Foundation Patient Registry from 2003 to 2013, including 3340 adult patients with a FEV_1_ < 30% prior to lung transplant. Median transplant-free survival was 6.6 years. There was substantial heterogeneity in survival, with some patients with CF dying soon after reaching this lung function threshold and others living for many years.

Another study reported a relationship between early mortality and FEV_1_ < 30% of predicted and elevated PaCO_2_ > 50 mmHg [16]. In a 5-year survival model, another study evaluated the impact of different variables on survival and correlated it with a change in the FEV_1_ percentage predicted. Female sex, diabetes mellitus, *B. cepacia* infection, and the number of exacerbations negatively affected the survival of patients with CF, whereas FEV_1_ percentage predicted alone was not a sufficient predictor of early mortality [17].

The importance of heterogeneity of the disease and inter-population variations in determining the survival in adult CF patients in different populations was highlighted by a recent report [4]. In contrast to these previous studies, in the current study we demonstrated that the median survival (37 months) was below that expected. We could hypothesize that some of the factors related with this worse prognosis are late diagnosis, poor socioeconomic conditions and genetic aspects [18]. It is suggested that further studies are conducted with other larger samples and including other regions of Brazil.

Prior studies evaluating survival in patients with CF have also documented increased risk of death among patients with lower BMI [11, 14, 19]. Ramos et al. [11] observed a high mortality associated with a BMI < 18 kg/m^2^.

In Brazil, the public health system provides ambulatory and hospital health carefree of charge. Also, CF medications are available through the public health system known as SUS (Sistema Único de Saúde) entirely free of any cost. Some CF medications are part of a protocol of medications that have been approved by Brazilian Health Ministry. The Health Secretary of each state is responsible for the delivery of the medications through public health care units or through public pharmacies. Usually, the CF care (CF Centers) is performed in each state in public hospital or university hospital. During the period of the study, the following medications were available: alfa dornase, colistin, tobramycin solution for inhalation, pancreatic enzymes, fat-soluble vitamins (A, D, E and K), ursodeoxicolic acid, azithromycin, dietary supplements, oral antibiotics for pulmonary infectious exacerbation. During the period of the study, new therapies such ivacaftor and ivacaftor/lumacaftor had not yet been approved in Brazil. State of Rio Grande do Sul, in southern Brazil, has a total area of around 281,748 km^2^ and 11.29 million inhabitants. There are three CF centers in Rio Grande do Sul, all of them located in the capital Porto Alegre. Almost all patients attending the Adult CF Centre of HCPA were from were living in the state of Rio Grande do Sul.

In Brazil there are six lung transplantation centers, two of them located in Porto Alegre. In Brazil, lung transplantation candidates are listed according to the chronological criterion. When there is organ supply, active patients compete according to waiting list time, provided there is ABO blood group identification, ribcage size compatibility, updated immunological reactivity test and, eventually, ABO compatibility (in the absence of receptors with the same ABO blood group as the donor). Patients who receive an organ that does not function within the first 48 h are an exception to the criteria above. In these situations, these receptors can be listed to receive another organ with urgency, being automatically placed at the top of the list [20]. The mean time on the waiting list for lung transplantation during the period of the study was approximately 18 months [21].

Guidelines recommend tobramycin solution for inhalation in patients over 6 years with chronic *P. aeruginosa* [22]. Colistin is used widely in Europe to treat chronic bacterial infection with *Pseudomonas aeruginosa*. In Brazil, inhaled colistin therapy is less expensive then inhaled tobramycin [23]. So, during the period of the study, in our institution the first option to treat chronic infection with *P. aeruginosa* was colistin. We reserved the use of inhaled tobramycin for patients with advanced lung disease. Consequently, patients who receive inhaled tobramycin in our service are more likely to have worse disease severity. The association of tobramycin inhalation with time to death in the present study could interpreted as confounding by severity [24].

The present study has some potential limitations. First, the investigation was done in a single centre. Second, the sample size was too small and would be associated with low statistical power. Mainly, because too many variables entered Cox regression models. Third, the retrospective study design did not allow us to assess some potential risk factors for mortality (parameters that were not collected).

The clinical relevance of the present study is to alert Brazilian adult CF physicians to the lower survival rate of adult CF subjects with FEV_1_ < 30% of predicted. Also, the association with a long time in lung transplant waiting list, should suggest that early referral for consideration of lung transplantation is highly desirable.

## Conclusion

In conclusion, we demonstrated that the median survival of CF subjects with FEV_1_ < 30% of predicted was 37 months. The main factors associated with time to death while in the study were BMI and tobramycin inhalation therapy. The best cut-off point selected for BMI was 18.5 kg/m^2^. Mean survival in patients with BMI < 18.5 kg/m^2^ was significantly lower (39.9 months) than in patients with BMI ≥ 18.5 kg/m^2^ (65.5 months). It is hoped that targeting these individuals for more intense nutritional optimisation will improve the survival.

## Data Availability

The datasets supporting the conclusions of this article are included within the article and its additional files. The statistical database may be available upon request.

## Abbreviations

CF: Cystic fibrosis
FEV_1_: Forced expiratory volume in one second
HCPA: Hospital de Clínicas de Porto Alegre
CFTR: Cystic fibrosis transmembrane conductance regulator
BMI: Body mass index
CFRD: Cystic fibrosis related diabetes
ABPA: Allergic bronchopulmonary aspergillosis
DIOS: Distal intestinal obstruction syndrome (DIOS)
MRSA: Methicillin resistant *Staphylococcus aureus*
NTM: Nontuberculous mycobacteria (NTM)
PASP: Pulmonary artery systolic pressure
6-MWT: 6-min walk test
HR: Hazard ratios
RR: Relative risk
ROC: Receiver operating curve
CI: Confidence interval
FVC: Forced vital capacity
SpO_2_: Saturation of peripheral oxygen
ICU: Intensive care unit
